# Abce1 orchestrates M-phase entry and cytoskeleton architecture in mouse oocyte

**DOI:** 10.18632/oncotarget.16546

**Published:** 2017-03-24

**Authors:** Xiao-Fei Jiao, Chun-Jie Huang, Di Wu, Jia-Yu Zhang, Yu-Ting Long, Fan Chen, Xiang Li, Li-Jun Huo

**Affiliations:** ^1^ Key Laboratory of Agricultural Animal Genetics, Breeding and Reproduction, Education Ministry of China, Wuhan, China; ^2^ College of Animal Science and Technology, Huazhong Agricultural University, Wuhan, China

**Keywords:** abce1, mouse oocyte, spindle assembly, chromosome alignment, aneuploidy

## Abstract

ATP-binding cassette E1 (ABCE1) is a member of the ATP-binding cassette transporters and essential for diverse biological events regulating abroad range of biological functions including viral infection, cell proliferation, anti-apoptosis, translation initiation and ribosome biogenesis. Here, we discovered that Abce1 also plays indispensable roles in mouse oocyte meiotic progression. In the present study, we examined the expression, localization, and function of Abce1 during mouse oocyte meiotic maturation. Immunostaining and confocal microscopy identified that Abce1 localized as small dots in nucleus in germinal vesicle stage. After germinal vesicle breakdown, it dispersedly localized around the whole spindle apparatus. During the anaphase and telophase stages, Abce1 was just like a cap to localize around the two pole region of spindle but not the midbody and chromosome. Knockdown of Abce1 by specific siRNA injection delayed the resumption of meiosis (GVBD) and affected the extrusion of first polar body. Moreover, the process of spindle assembly and chromosome alignment were severely impaired. Abce1-RNAi led to the dissociation of γ-tubulin and p-MAPK from spindle poles, thus caused mounts of spindle morphology abnormities and chromosome alignment defects, leading to high incidence of aneuploidy. Our findings refresh the knowledge of Abce1 function by exploring its role in oocyte meiotic resumption, spindle assembly and chromosome alignment.

## INTRODUCTION

In mammals, oocyte employs two extremely asymmetric divisions, meiosis I and meiosis II, with only one round of DNA replication thereon yielding the egg, a highly polarized gamate, which can wait the sperm for fertilization and execute a molecular program for development [1]. Chromosome alignment and segregation occur on a spindle-shaped structure that is built from microtubules. Defective structure of spindle and abnormal segregation of chromosome in meiosis could bring on aneuploidy. Mammalian oocytes are prone to chromosome segregation errors which can lead to aneuploid fetuses [2, 3]. Most embryonic aneuploidies in humans are incompatible with development, as for this reason, fetal aneuploidy is thus a major cause of pregnancy loss [4, 5]. To ensure orderly meiosis during oocyte maturation, spindle assembly and chromosome alignment must be accurately controlled.

When immature oocytes begin to mature by hormonal stimulation, germinal vesicle breakdown (GVBD) happens and meiotic spindle formation is well completed at metaphase I (MI) stage [6]. In many animals, including nematodes, insects, and vertebrates, oocyte meiotic spindles assemble without the function of canonical centrosomes which govern bipolar spindle assembly during mitosis. When mitosis starts going on, at least two distinct pathways function in microtubule nucleation during spindle assembly, one centrosome dependent and the other chromosome dependent [7]. Motor proteins and other microtubule-associated proteins, but not centrosomes, orchestrate bipolar spindle assembly during meiosis [8]. Spindle microtubules in intact vertebrate oocytes originate from Microtubule Organizing Centers (MTOCs) comprising γ-tubulin and additional microtubule nucleators, including phospho-MAPK [9, 10]. During cell division, bipolar spindle microtubules can gather and sort chromosomes with the help of associated proteins involved in this special progress and then allocate the forces necessary to dispatch copies of replicated chromosomes to daughter cells [11].

The ABCE1 protein is a member of the superfamily of ATP binding cassette (ABC) proteins which was originally identified for its inhibition of ribonuclease L and is composed of two nucleotide binding domains and an N-terminal Fe-S binding domain. ABCE1 functions in translation initiation, ribosome biogenesis, cell proliferation, viral infection, anti-apoptosis and human immunodeficiency virus capsid assembly [12]. Recent studies have also determined the associations of ABCE1 with varieties of tumorigenesis [13–16].

Oocyte maturation is a complex and precisely synchronized process affected by many factors, whether Abce1 takes part in the control of mouse oocyte meiosis is unknown. By employing siRNA knockdown analysis, we discovered the involvement of Abce1 in meiosis of mouse oocyte, especially in controlling meiotic progression and spindle morphology.

## RESULTS

### Abce1 localization and expression during mouse oocyte meiotic maturation

**Figure 1:**
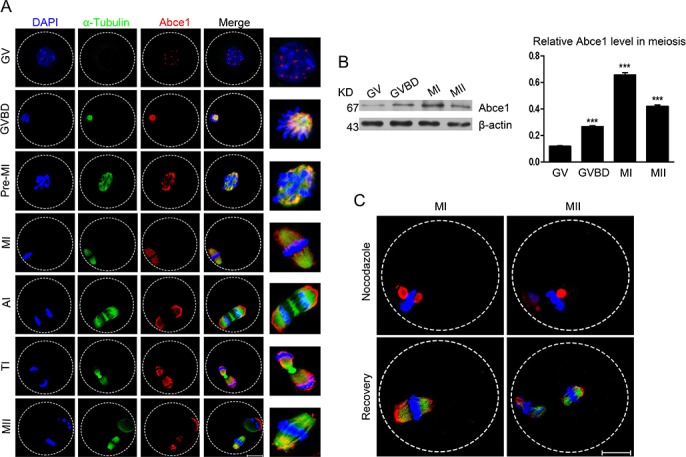
Cellular localization and expression of Abce1 during mouse oocyte maturation **(A)** Cellular localization of Abce1 detected by immunofluorescent analysis. Oocytes at indicated stages were immunostained for Abce1 (red), microtubule (α-tubulin; green) and DNA (blue). Magnification of the boxed regions showed relationship of Abce1 with the spindle. **(B)** Expression of Abce1 during mouse oocyte meiotic maturation. Oocytes were collected after 0, 2, 8, or 14 h in culture, corresponding to GV, GVBD, MI, and MII stage, respectively. The molecular weight of Abce1 and β-actin were 67 kD and 43 kD, respectively. Normalized signal intensity of Abce1 was presented in the right panel. **(C)** Confocal images of Abce1 signal after treatment with nocodazole. Oocytes at indicated stage were double stained for Abce1 (red), α-tubulin (green) and DNA (blue). Data were presented as mean percentage (mean ± SEM) of at least three independent experiments. ***p < 0.001. Scale bar, 20 μm.

To investigate the role of Abce1 in mouse oocyte maturation, we first examined the dynamic distribution and expression of Abce1 at different stages. Oocytes were cultured *in vitro* for 0, 2, 4.5, 8, 9.5 or 14 h until they reached the GV, GVBD, Pre-MI, MI, ATI, and MII stages, respectively. The subcellular distribution of Abce1 during mouse oocyte meiotic maturation was examined by confocal immunofluorescence microscopy. As shown in Figure 1A, Abce1 localized as big dots in the germinal vesicle. Shortly after GVBD, Abce1 accumulated around the chromosome region and co-localized with α-tubulin. During the Pre-MI, MI and MII stages, Abce1 localized around the whole spindle apparatus. Specifically, Abce1 was just like a cap to localize around the two pole region of spindle but not the midbody and chromosome during the anaphase and telophase stages. We next performed western blotting to determine the expression level of Abce1 during mouse oocyte meiotic maturation. About 200 oocytes were used and the result showed that the expression of Abce1 in oocytes significantly increased from GV to MI stage and later slightly declined in MII stage. (Figure 1B). To verify the localization pattern of Abce1 in mouse oocyte meiosis, we treated MI and MII oocytes with nocodazole, a microtubule-depolymerizing agent. After treatment, the microtubules were completely disassembled, and no intact spindles were observed in oocytes. Unexpectedly, Abce1 staining did not disperse into the cytoplasm as the microtubules did, big dots occurred around the chromosomes. Furthermore, when the nocodazole-treated oocytes were thoroughly washed and cultured in nocadazole-free medium to allow microtubule re-assembly, Abce1 renewed its original localization, implying Abce1 did not localize on microtubules but depend on the shape of spindle to localize (Figure 1C).

### Knockdown of Abce1 delays the resumption of meiosis (GVBD) and affects first polar body extrusion

**Figure 2:**
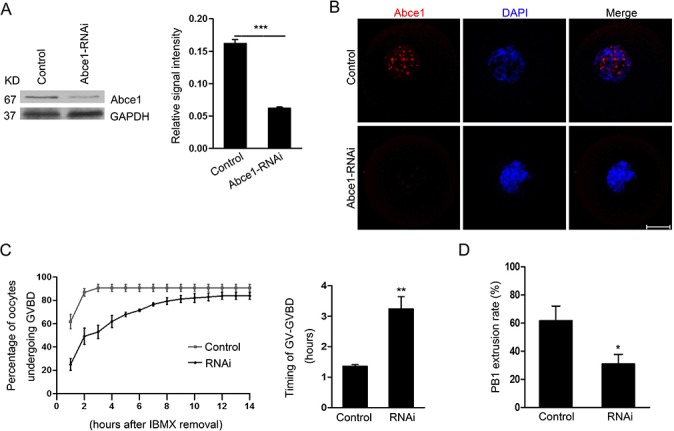
Abce1 RNAi delays the resumption of meiosis (GVBD) and affects first polar body extrusion Oocytes injected with control or Abce1 siRNA were incubated in M2 medium containing 50 μM IBMX for 24 h and then released into IBMX-free M16 medium for continuously culture. **(A)** Western blot showing the reduced expression of Abce1 after siRNA injection. Abce1 relative expression level was normalized to values found in control oocytes (right panel). **(B)** Knockdown efficiency of Abce1 protein was validated by immunostaining of Abce1 (red) and DNA (blue). **(C)** The kinetics of GVBD was scored at different time points as indicated in the graph, the transition timing of GV to GVBD was measured and shown in the right panel. **(D)** Characterization of polar body extrusion (PBE) rate after 14 h in culture. A total of 215 oocytes in control and 222 oocytes in Abce1-RNAi were analyzed. Data were presented as mean percentage (mean ± SEM) of at least three independent experiments. *p < 0.05 and ***p < 0.001. Scale bar, 20 μm.

To assess its function, Abce1 was knocked down by microinjection of Abce1 siRNA. Compared with oocytes microinjected with control siRNA (control), western blot and immunostaining revealed that the expression of Abce1 was significantly reduced in oocytes microinjected with Abce1 siRNA (Figure 2A and 2B). We then analyzed the rate of meiotic resumption *in vitro*, characterized by the disappearance of the nucleus (also named germinal vesicle breakdown (GVBD)). We found that, knockdown of Abce1 significantly delayed the resumption of meiosis (GVBD) and the transition GV-GVBD occurred on average after 1.36 ± 0.05 hours for control oocytes compared with 3.24 ± 0.40 hours for Abce1 siRNA-injected oocytes (Figure 2C). After 14h in culture, most microinjected oocytes progressed through meiotic resumption, but only 30.8 ± 6.79% of these oocytes extruded the polar body (Figure 2D), in comparison to 61.6 ± 10.52% of control oocytes.

### Knockdown of Abce1 causes defective spindle morphogenesis and abnormal chromosome alignment

**Figure 3:**
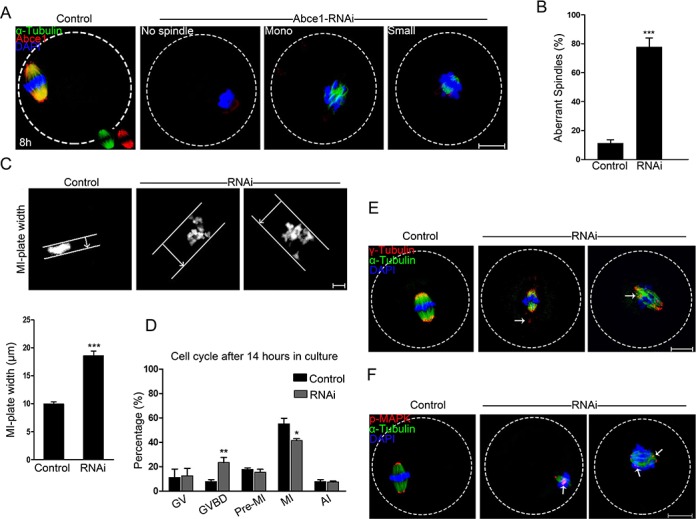
Knockdown of Abce1 causes defective spindle morphogenesis and abnormal chromosome alignment **(A)** Spindle morphology of control and Abce1-RNAi oocytes after 8 h in culture. Control oocytes present a bipolar barrel-shaped spindle, whereas spindle defects were frequently observed in Abce1-RNAi oocytes. Representative confocal sections are shown. Scale bar, 20 μm. **(B)** Quantification of control (n=108) and Abce1-RNAi (n=87) oocytes with abnormal spindles. Scale bar, 20 μm **(C)** Metaphase I (MI) plate width was determined by measuring the axis distance between the two lines at the edges of the DNA. Scale bar, 10 μm. Quantification of metaphase I plate width in control and Abce1-RNAi oocytes were presented in the lower panel. At least 30 oocytes were analyzed in control and RNAi group. **(D)** Graph showing the cell cycle analysis of un-matured oocytes in control (n=133) and Abce1-RNAi group (n=121) after 14 h in culture. **(E)** Representative images of MI oocytes stained with anti-γ-tubulin (red), α-tubulin (green) antibodies and DAPI (blue). γ-tubulin displayed mislocalization (arrows) in Abce1-RNAi oocytes instead of concentrating on spindle poles as in control oocytes. Scale bar, 20 μm. **(F)** Images are MI oocytes stained with anti-p-MAPK (red), α-tubulin (green) antibodies and DAPI (blue). Notably, p-MAPK in control oocytes appeared at the spindle poles; conversely, disrupted localization (arrows) was detected in Abce1-RNAi oocytes. Scale bar, 20 μm. Data were presented as mean percentage (mean ± SEM) of at least three independent experiments, *p < 0.05, **p < 0.01 and ***p < 0.001.

Because of a prominent polar body extrusion decline in RNAi oocytes, we next examined the spindle assembly in oocytes after Abce1 knockdown. Confocal microscopy revealed that most control oocytes at metaphase I presented with a typical barrel-shape spindle, while knockdown of Abce1 even vanished spindle apparatus in Abce1-RNAi oocytes and most of them displayed diverse malformed spindles (Figure 3A). The proportion of oocytes with abnormal spindles in Abce1 knockdown oocytes was significantly higher than in the control group (77.73 ± 6.23% vs. 11.25 ± 2.37%, *p* < 0.001; Figure 3B). The alignments of chromosomes were also checked, whereas chromosomes were well-aligned at the metaphase I plate in control oocytes, Abce1 knockdown oocytes exhibited an increased incidence of chromosome misalignment (Figure 3C). Cell cycle analysis of those un-matured oocytes (post 14 h in culture) in Abce1-RNAi group revealed that the rate of oocytes arrested at GVBD stage was significantly higher than that in the control group (Figure 3D), which was consistent with the result that Abce1 knockdown can delay the GVBD.

To illuminate the underlying mechanism for defective spindle morphogenesis in Abce1 knockdown oocytes, γ-tubulin, a well-recognized MTOC-associated protein regulating spindle morphogenesis was examined. In control MI oocytes, γ-tubulin localized at the spindle poles. Conversely, Abce1-RNAi markedly distorted the localization of γ-tubulin (Figure 3E). Additionally, Phospho-MAPK, another important microtubule nucleator factor was also analyzed in our study. Of note, in striking contrast to control MI oocytes in which phospho-MAPK localized at the spindle poles, the localization of phospho-MAPK in Abce1-RNAi oocytes was distorted with dispersed signals around spindles (Figure 3F). Taken together, we could contend that γ-tubulin and phospho-MAPK dysfunctions were involved in spindle morphogenetic defects induced by Abce1 knockdown.

### Abce1 knockdown causes activation of the spindle assembly checkpoint

**Figure 4:**
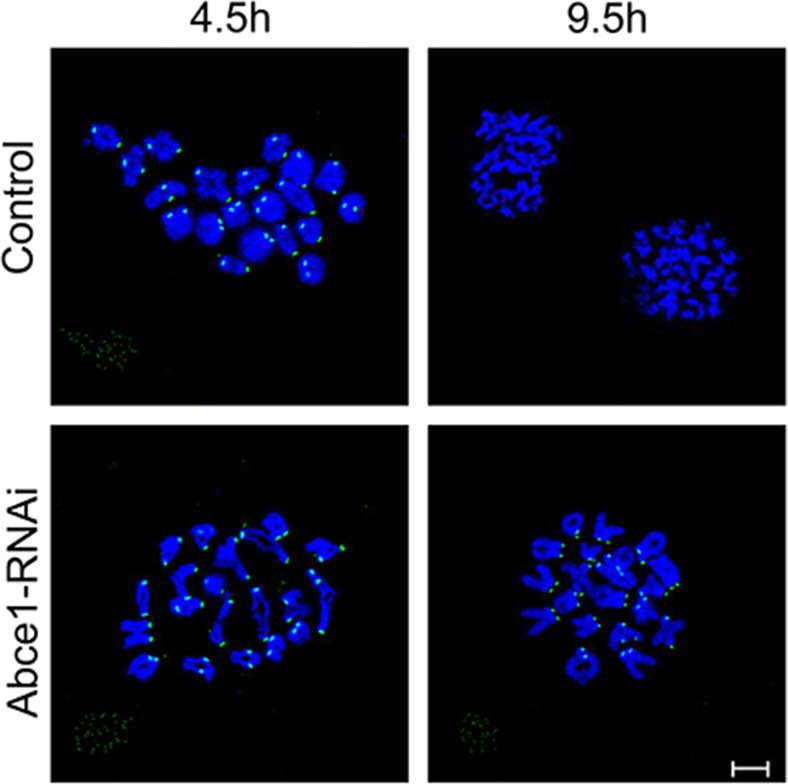
Abce1 knockdown causes activation of the spindle assembly checkpoint After microinjection of Abce1 siRNA or control siRNA, the oocytes were placed in M16 containing IBMX for 24 h, then washed 5 times and transferred into IBMX-free M16 medium for 4.5h and 9.5h, corresponding to Pre-MI and AI stage, followed by chromosome spreading of BubR1. BubR1, green; DNA, Blue. Bar, 10 μm.

Defective spindle morphogenesis and abnormal chromosome alignment can cause the activation of spindle assembly checkpoint (SAC) which may lead to the failure of polar body extrusion. Sever defects of spindle morphogenesis and chromosome alignment were observed in Abce1 knockdown oocytes which motivated us to determine the activity of spindle assembly checkpoint (SAC). We analyzed BubR1 in oocytes, which is a principal component of SAC. The BubR1 signals at kinetochores were comparable in both groups at pro-MI stage (4.5 h). When oocytes proceed to anaphase I (9.5 h), homologous chromosomes were segregated and the localization of BubR1 disappeared from kinetochores in control oocytes. However, in Abce1 knockdown oocytes, BubR1 signals still persisted at kinetochores and the homologous chromosomes failed to segregate (Figure 4), indicating the activation of the SAC.

### Increased incidence of aneuploidy in Abce1 knockdown oocytes

**Figure 5:**
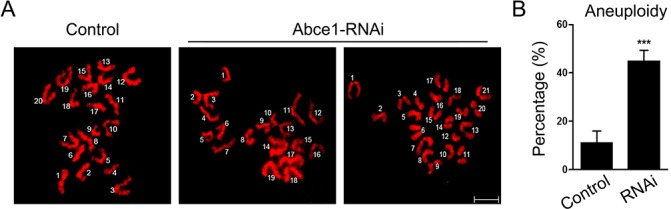
Increased incidence of aneuploidy in Abce1 knockdown oocytes **(A)** Chromosome spread of control and Abce1-RNAi MII oocytes. Chromosomes were stained with DAPI (red). Representative confocal images indicate control oocytes with a normal haploid complement of 20 chromosomes, Abce1-RNAi oocytes with 19 and 21 chromosomes. **(B)** Quantification of aneuploidy in control and Abce1-RNAi oocytes. 30 control oocytes and 31 Abce1-RNAi oocytes were analyzed respectively. Data were presented as mean percentage (mean ± SEM) of at least three independent experiments, ***p < 0.001. Bar, 10 μm.

Since Abce1 knockdown led to spindle and chromosome anomalies, we postulated that aneuploid eggs would be generated from Abce1-RNAi oocytes. To test this hypothesis, we analyzed the karyotype of MII oocytes by chromosome spreading. As shown in Figure 5A, the number of single chromosomes (univalents) in the normal eggs was 20, which is expected in the mouse for genomic integrity. Whereas a significantly higher incidence of aneuploid eggs that had more or less 20 univalents was found in Abce1 knockdown oocytes compared to controls (44.84 ±4.51% vs. 11.11 ± 4.81%, *p* < 0.001; Figure 5B). In conclusion, loss of Abce1 impairs the assembly of meiotic spindle and accurate chromosome movement, therefore, elevating the incidence of aneuploidy.

## DISCUSSION

Errors in chromosome segregation and spindle defects in oocytes lead to embryo aneuploidy, which contributes to early pregnancy loss. However, under-standing the regulation of these two events in the absence of typical centrosomes in mammalian oocytes has not been studied as well as in mitotic somatic cells. In the present study we discover a novel role for Abce1 during meiosis: the involvement of cell cycle progression and spindle integrity in meiotic mouse oocytes.

To find out the relevance of Abce1 in meiotic program, we firstly studied the localization and expression patterns of Abce1 during mouse oocyte meiotic progression. Immunofluorescent analysis showed that Abce1 predominantly distributed in nucleus as big dots in immature oocytes. Microtubule-associated dynamic of Abce1 occurred with meiotic resumption. To further confirm the localization pattern of Abce1, nocodazole treatment was conducted. When oocytes were exposed to nocodazole to disassemble microtubules, Abce1 staining did not disperse into the cytoplasm, instead big dots occurred around the chromosomes, suggesting Abce1 did not localize on microtubules. During the anaphase and telophase stages, Abce1 was just like a cap to localize around the two pole region of spindle but not the midbody and chromosome. The expression profile of Abce1 during oocyte meiosis was also consistent with previous oocyte proteomic study [17]. Knockdown of Abce1 delayed the onset of germinal vesicle breakdown and caused abnormal spindle assembly with the defective chromosome alignment accompanied.

Spindle apparatus which is built from microtubules, 25 nm diameter tubes composed of α/β-tubulin dimers that host the alignment and segregation of chromosomes. There are so many microtubule-associated proteins and accurate protein modifications needed to modulate microtubules organizing into a bipolar spindle-shaped structure [8, 18–21]. Centrosome was found to be the primary MTOC in animal somatic cells, whereas non-centrosomal MTOCs were used to build the bipolar spindle as the centrosome did in the progress of mitosis. In spite of their different shapes and sizes, all MTOCs depend on γ-tubulin and additional microtubule nucleators including phospho-MAPK whose function in modulating meiotic spindle has already been identified in mouse oocyte to nucleate microtubules [8–10, 22]. We found that knockdown of Abce1 led to the dissociations of γ-tubulin and phospho-MAPK from spindle poles, indicating that Abce1 may play a role in spindle organization by maintaining the recruitment of γ-tubulin and phospho-MAPK to the pole region.

Anomalies in spindle assembly and chromosome alignment would lead to the generation of aneuploid eggs because of unfaithful segregation of chromosomes. There is a key regulator of chromosome segregation named spindle assembly checkpoint (SAC) [23–25]. Mad (mitotic-arrest deficient) proteins and Bub (budding uninhibited by Benz imidazole) proteins are important SAC proteins to play indispensable roles in supervising proper chromosome segregation [26, 27]. These proteins accumulate at kinetochores to prevent precocious anaphase onset before chromosomes have achieved proper bipolar attachment to the spindle during mitosis. SAC inhibits the ability of APC/C^Cdc20^ to target substrates for degradation and then prevent anaphase onset [28, 29]. Unlike its role in mitosis progression, although SAC functions in mammalian oocytes, meiosis I (MI) is still error prone and polar-displaced chromosomes do not block anaphase onset [30]. Abnormal chromosome alignment and segregation easily cause high incidence of aneuploidy. BubR1, a member of SAC, plays an important role in regulating mouse oocyte meiotic anaphase entry [31]. In this study, BubR1 was used to detect whether the SAC is provoked because of the defective spindle morphogenesis and abnormal chromosome alignments. In contrast to control oocytes, BubR1 signals were vanished from kinetochores after 9.5 h in culture, while it persisted at kinetochores in Abce1-RNAi oocytes, confirming that knockdown of Abce1 provokes the SAC, thus may cause MI phase arrest and/or delayed anaphase onset. In addition, there were about 30% of Abce1-RNAi oocytes still extruded the first polar bodies although with high incidence of aneuploidy accompanied, suggesting knockdown of Abce1 did not totally block APC/C activity.

In summary, our study pinpoints that Abce1 plays an important role in meiosis resumption, spindle integrity and chromosome alignment in meiotic oocytes. Further studies are needed to elucidate the precise mechanism and biological significance of Abce1 in oocyte maturation.

## MATERIALS AND METHODS

### Antibodies and reagents

Rabbit anti-ABCE1 monoclonal antibody (Cat# 185548) and sheep anti-BubR1 polyclonal antibody (Cat# 28193) were obtained from Abcam (Cambridge, UK); rabbit anti-phospho-p44/42 MAPK monoclonal antibody (Cat# 4370) was purchased from CST (Danvers, MA); mouse anti-γ-tubulin monoclonal antibody (Cat# 17787) was purchased from Santa Cruz (Santa Cruz, CA); mouse monoclonal anti-α-tubulin-FITC antibody (Cat# F2168) was obtained from Sigma (St Louis, MO). FITC-conjugated donkey anti-sheep IgG (H + L) was produced by Jackson ImmunoResearch Laboratories, Inc. Cy3-conjugated goat anti-rabbit IgG (H + L) and Cy3-conjugated goat anti-mouse IgG (H + L) were purchased from Boster Biotechnology Co., LTD (wuhan, China)

.

Spindle perturbation drug nocodazole was 10 mg/ml in DMSO stock (−20°C), was purchased from Sigma-Aldrich Co. All other reagents were purchased from Sigma Aldrich unless specifically stated otherwise.

### Animals and ethics statement

Kunming strain (KM) mice were obtained from local Central Animal Laboratory and were bred at the experimental animal center of Huazhong Agricultural University under a 12 h light/dark cycle with water and food ad libitum. This study was approved by the Ethical Committee of the Hubei Research Center of Experimental Animals (Approval ID: SCXK (Hubei) 20080005). All experimental protocols were conducted in accordance with the guidelines of the Committee of Animal Research Institute, Huazhong Agricultural University, China.

### Oocyte collection, culture and drug treatment

Ovaries were isolated from 3-4 week-old KM mice. 48 hours after pregnant mare serum gonadotropin (PMSG) injection, cumulus-oocyte complexes were collected by manual rupturing of antral ovarian follicles. To obtain fully grown GV oocytes, cumulus cells were removed by repeatedly pipetting and oocytes were collected in pre-warmed (37°C) M2 medium supplemented with 50 μM IBMX to arrest the oocytes at GV-stage. To induce meiotic maturation, oocytes were washed out of IBMX and cultured in M16 medium for 0, 2, 4.5, 8, 9.5 and 14 h, corresponding to GV stage, germinal vesicle breakdown (GVBD) stage, pre-metaphase I (Pre-MI), metaphase I (MI), anaphase I (AI)/telophase I (TI) and metaphase II (MII), respectively. After specific periods of culture, oocytes were collected for GVBD or PBI (MII) observation, drug treatment, microinjection, western blotting or immunofluorescent analysis.

For drug treatment, wide-type MI or MII stage oocytes were cultured in M16 medium containing 20 μg/ml of nocodazole for 15 min, followed by immunostaining of Abce1 and α-tubulin. For microtubule re-assembly, after incubation with nocodazole for 15 min, oocytes were then washed thoroughly and recovered in fresh M16 medium for 30 min, and collected for immunofluorescent analysis. All control oocytes were cultured in M16 medium containing the same concentration of DMSO.

### Immunofluorescence and confocal microscopy

Specific stage of oocytes were briefly washed through PHEM solution (60 mM PIPES at pH 6.9, 25 mM HEPES, 10 mM EGTA, 2 mM MgCl_2_.7H_2_O) and fixed using 4% paraformaldehyde in PHEM containing 0.5% Triton X-100 for 30 min. Oocytes were then blocked in PBS containing 2% BSA and 0.05% Tween-20 for 1 h at room temperature and incubated with proper primary antibodies overnight at 4°C. After washing in PBS containing 0.05% Tween-20 for 3 times and 10 min each, oocytes were incubated with the corresponding secondary antibodies for 1 h at 37°C. For double-staining, after secondary antibody incubation, oocytes were blocked again in blocking solution for 1 h at room temperature, and then incubated with the other primary antibodies, oocytes were processed with the secondary antibodies corresponding to the second primary antibodies. DNA was labelled in PBS containing 1 μg/ml of DAPI for 10 min at room temperature. Finally, oocytes were mounted on glass slides with DABCO and examined with a confocal laser scanning microscope (Zeiss LSM 510 META, Carl Zeiss Imaging, Germany) equipped with a Plan-Apochromat 63×/1.4 oil DIC objective. Confocal images were processed using Zeiss LSM Image Browser software and Adobe Photoshop (Adobe Systems Inc., San Jose, CA).

For immunolabelling, the following primary antibodies and dilutions were used: rabbit anti-ABCE1 antibody (1:100), mouse anti-γ-tubulin antibody (1:50), rabbit anti-phospho-p44/42 MAPK monoclonal antibody (1:100) and FITC-labelled mouse anti-α -tubulin (1:100). Cy3- labelled goat anti-rabbit or goat anti-mouse (Boster; 1:100) antibodies were used as the secondary antibodies.

### Immunoblotting

About 200 oocytes each group were briefly washed in PBS and then lysed in 2 × SDS sample buffer and stored at −80°C until use. For blotting, samples were heated at 100°C for 5 min and then placed on ice for 5 min. The proteins were separated by SDS-PAGE and electri-cally transferred to PVDF membranes (Immobilon-P; Millipore). The membranes were blocked in 5% BSA in TBS (25 mM Tris, 150 mM NaCl, pH 8.0) containing 0.01% Tween-20 (TBST) for 1 h and then incubated overnight at 4°C with the primary antibodies: rabbit monoclonal anti-ABCE1 (1:1000). After washing with TBST, secondary antibodies were used as anti-rabbit HRP conjugates (1:3000; Boster), the immunoblot bands were visualized with ECL kit and read using chemiluminescence system (Thermo Scientific). Beta-actin (Santa Cruz, 1:500) and GAPDH (Santa Cruz, 1:500) were served as a loading control. The relative signal intensity was assessed by Image J software (NIH, USA).

### Knockdown of Abce1 by siRNA microinjection

For microinjection, GV-stage oocytes were collected in M2 medium containing 50 μM IBMX, and 5–10 pL of 30 μM control siRNA (sc-37007; Santa Cruz, CA) or Abce1 siRNA (sc-60118; Santa Cruz, CA) was injected into the cytoplasm of oocyte. Following microinjection, oocytes were cultured in M2 medium supplemented with 50 μM IBMX for 24 h to achieve Abce1 knockdown. After that, oocytes were directly collected for western blotting or immunostaining or thoroughly washed out of IBMX and released into M16 medium for meiotic maturation or other experiments.

### Chromosome spreading

Chromosome spreading was performed as described previously [32]. Briefly, oocytes were exposed to Tyrode's buffer (pH 2.5) for about 30 s at 37°C to remove zona pellucidae. After recovery in M2 medium for 10 min, oocytes were fixed in a drop of 1% paraformaldehyde with 0.15% Triton X-100 on a glass slide. After air drying, slides were washed and blocked with PBS containing 2% BSA, followed by incubating with sheep anti-BubR1 antibody (1:100) and FITC-conjugated donkey anti-sheep antibody (1:100) and DNA was visualized by DAPI staining. Finally, the slides were mounted and examined with the confocal laser scanning microscope.

### Statistical analysis

Data from at least three independent replicates was presented as mean ± SEM and analyzed by paired-samples t-test using SPSS software (SPSS Inc, Chicago, IL) with *p* < 0.05 was considered to be statistically significant. Different superscripts indicate the statistical difference.
